# Monitoring oral health remotely: ethical considerations when using AI among vulnerable populations

**DOI:** 10.3389/froh.2025.1587630

**Published:** 2025-04-14

**Authors:** Colman McGrath, Chun Wang Reinhard Chau, Gustavo Fabián Molina

**Affiliations:** ^1^ Applied Oral Sciences and Community Dental Care Division, The Faculty of Dentistry, University of Hong Kong, Hong Kong, Hong Kong SAR, China; ^2^ Special Care Dentistry, School of Dentistry, Universidad Católica de Córdoba, Cordoba, Argentina

**Keywords:** artificial intelligence, special care dentistry, digital dentistry, ethics, oral health inequalities

## Abstract

Technological innovations in dentistry are revolutionizing the monitoring and management of oral health. This perspective article critically examines the rapid expansion of remote monitoring technologies—including artificial intelligence (AI)-driven diagnostics, electronic health records (EHR), wearable devices, mobile health applications, and chatbots—and discusses their ethical, legal, and social implications. The accelerated adoption of these digital tools, particularly in the wake of the COVID-19 pandemic, has enhanced accessibility to care while simultaneously raising significant concerns regarding patient consent, data privacy, and algorithmic biases. We review current applications ranging from AI-assisted detection of dental pathologies to blockchain-enabled data transfer within EHR systems, highlighting the potential for improved diagnostic accuracy and the risks associated with over-reliance on remote assessments. Furthermore, we underscore the challenges posed by the digital divide, where disparities in digital literacy and access may inadvertently exacerbate existing socio-economic and health inequalities. This article calls for the development and rigorous implementation of ethical frameworks and regulatory guidelines that ensure the reliability, transparency, and accountability of digital health innovations. By integrating multidisciplinary insights, our discussion aims to foster a balanced approach that maximizes the clinical benefits of emerging technologies while safeguarding patient autonomy and promoting equitable healthcare delivery.

## 1 Introduction

Technological innovations in medicine and dentistry have led to the rapid development in the possibility of monitoring health and oral health remotely (1, 2). This has had a long history stemming from how to manage oral health in remote communities and for those requiring special care dentistry where routine access in primary care can be an issue (3, 4). There has been an exponential acceleration of such technologies since COVID and particularly during periods of lockdown (5, 6), and these technologies continue to unfold but are not without ethical concerns (7, 8).

There are many potential benefits of monitoring health and oral health remotely in terms of (i) easier access to care for diagnoses and easier access to advice as in the case of oral medicine, especially in remote areas or where there are difficulties in accessing care in usual ways which can be an issue in aged care homes (1); (ii) monitoring oral health remotely has the potential for faster and more convenient access to care resulting in a reduction of loss of work time and travel cost for patients, and this can be considerable saving where there are inequitable distribution of services geographically, for example of specialist opinions (9); (iii) monitoring oral health remotely can lead to faster decision making on potential care pathways leading to delivery of appropriate care when needed to maximize effectiveness (10); (iv) achieving improved continuity of care with easier access to monitor outcomes of treatment (11, 12), this can be a considerable problem in low and middle income countries where follow-up in the absence of pain is a sizeable problem (13); and (v) monitoring oral heath remotely can potentially generate significant reduction in healthcare costs from a government provider, third party payer perspective with more efficient use of services (14). However, various legal and ethical concerns have emerged that warrant consideration.

The 21st century is characterized by a knowledge-and information-based society, where access to information and communication technology is essential for everyday life. Monitoring oral health remotely is part of this ever-growing movement. But a key issue is the ethics of this “digital divide”-the gap between individuals or communities who have access to information and communication technologies (ICT) and those who do not and, thereby, their ability to use such information (15). This is further compounded by the ability to find, evaluate, use, and create information from digital technologies.

These digital divide gaps have been reported to be based on factors such as socio-economic status, geographic location, age, education level, race and disabilities and thus, underserve already underserved communities (16, 17). To this end, the concept of Digital Determinants of Health has recently emerged because digital technology can exacerbate and reinforce pre-existing social health disparities (18). Digital determinants of health are implicit in the design of AI systems, mobile phone apps, telemedicine, digital health literacy, and other forms of digital technology (18). With the increasing use of digital technology in health care, the potential for health and oral health inequities and disparities must be addressed.

To overcome the digital divide, there is a need to promote a digitally inclusive society through internet commuter technologies and strategies that increase access to internet connectivity and technology, strengthen digital awareness, and improve digital literacy in oral health through training to reduce disparities and inequalities. Networking among health-related NGOs (especially those associated with vulnerable populations), governments and the third sector would benefit from alliances with exponential impact in the community, reaching further these target groups.

### 1.1 Digital health technologies in dentistry

AI-based technologies are decision systems based on clinical knowledge, which leads to helping and advising professionals in making better judgments. AI-based technology in Dentistry is a computer software created to assist professionals in making clinical choices. For that purpose, it is essential to input text, image or voice data so the software may produce a treatment, diagnosis or disease prediction output.

Examples of AI-based technology in Dentistry are briefly mentioned to introduce the topic. Artificial neural networks (ANN's) are systems based on algorithms that enable deep learning to analyze treatment planning, diagnosis, and prognosis prediction. Augmented reality/virtual reality is a simulation based on a 3D picture generated by the computer, which can interact with the real and physical world and electronic equipment, and may assist in treatment planning. Data mining is concerned with discovering causal links and similarities in current data, allowing the analysis of variance across dentists when detecting and predicting dental caries or dental age estimation. These technologies are adapted to the needs of dental disciplines such as Public Dental Health, Endodontics, Orthodontics, Prosthodontics, etc., to enhance diagnosis, treatment planning/execution and disease prediction (19).

Digital biomarkers are indicators of biologic processes, pathologic processes, or biological reactions to a therapeutic intervention that can be measured using digital devices such as smartphones, wearable devices, and Internet of Things (IoT) technologies (20). Mobile health (mHealth) technologies, used to input data, may facilitate oral disease prevention and management through early or timely identification of signs of pre-clinical or clinical functional deterioration, or by just providing oral health education. A literature review showed that mHealth could improve oral health management, oral health behavior, and oral health knowledge among older adults (1). Another review and metaanalysis concluded that teledentistry and mHealth are valuable tools for promotion and prevention of oral health (2). These technologies particularly apply to geographically and culturally remote groups such as aborigine communities or people living in rural villages (3, 4).

## 2 Discussion

### 2.1 Monitoring oral health remotely among vulnerable populations: concerning issues

#### 2.1.1 Obtaining consent

A recent scoping review of 17 studies published in 2024 identified 12 studies with ethical and legal issues related to patient consent in digital oral health (21).

The electronic informed consent differs from paper-based informed consent in ways that may impair the participant's autonomy to make decisions. Therefore, current constructs for electronic informed consent promote the engagement for comprehension, establish a suitable digital technology, ensure accessibility, give opportunity to gather information from parents or relatives, establish an appropriate language, ensure confidentiality and facilitate patientś autonomy to complete and/or modify their consent. Examples of best practices for e-consent are available in scientific papers, depending on the type of study or intervention that applies.

A key issue was that patients/participants should be fully informed about the nature of the remote monitoring technology and their diagnosis accuracy, often lower than traditional “in-person” oral health assessments (22). For the remote modality, patients are responsible for using and maintaining monitoring device procedures correctly, and this typically requires a certain level of technical proficiency that patients/participants need to be aware of and to be trained adequately to use (23).

Many issues surround how to obtain e-Consent with differing requirements by jurisdiction (24, 25). These e-Consent platforms often include interactive features that allow participants to navigate through the information at their own pace, access additional resources, and ask questions for clarification (26). This interactive approach promotes a better understanding of the requirements, potential risks and benefits.

Remote monitoring technologies may not be able to provide immediate assistance in emergencies (27); and users need to be aware of this, especially with the increased likelihood of urgent dental care (28). Likewise, remote technologies for diagnoses may have limited interface with care pathways or follow-up, and this raises the ethical issue of assessment and diagnosis without the means to provide the care (29, 30).

#### 2.1.2 Electronic health records (EHR)

We now have technologies that make it possible to digitize almost any type of dental information through electronic health records (EHR) so that it can be readily acquired, stored, retrieved, shared, and subsequently analysed (31). This has the potential to dismantle the traditional stand-alone dental records and promote a healthcare delivery system where providers in many disciplines can easily communicate within dentistry and healthcare systems.

Improving communication and interaction between medical and dental EHR will allow better synchronization and integration across healthcare systems (32). Moreover, EHR can improve overall quality by standardizing data collection with implications to advance clinical oral research, providing opportunities to improve oral health (33).

EHR raises many ethical issues, however. The issue of “Acquiring”, which was previously mentioned concerning patient consent, as well as issues of Storing, Retrieving and Sharing, are real potential ethical minefields (34). “Privacy and Confidentiality” are key issues since EHRs contain sensitive patient information, and maintaining the privacy and confidentiality of this data is of paramount importance (35). Unauthorized access, data breaches, and improper information sharing can compromise patient privacy and trust. Ensuring the security of EHRs is crucial to prevent data breaches, cyberattacks, and unauthorized access, and it is an ethical responsibility of the provider/organization to implement robust security measures, ensure encryption protocols are in place, and have access controls in place that protect patients' data from these threats (36).

A potential solution to the ethical issue of accessing, retrieving and sharing EHR has been proposed in the field of oral medicine, diagnosis and radiology (OMFR), which has longstanding experience in data transfer (37): Blockchain-enabled InterPlanetary File System (IPFS) (38).

Blockchain technology works as decentralized ledger technology with several key features: (i) Immutability – meaning that once data/records are written/uploaded to a blockchain, they cannot be altered or deleted, ensuring data integrity – thus, the historical records are preserved exactly as they were originally recorded, preventing any tampering or unauthorized modifications (39); (ii) The InterPlanetary File System (IPFS) handles the storage of files in a decentralized manner to maintain metadata and, thereby, a verifiable record of data stored, maintaining a secure ledger (40); (iii) Issues of access control and authentication: Smart contracts on the blockchain can manage access control to the files providing a secure and automated way so that only authorized users can access certain data (41); (iv) Blockchain can ensure that the references to data are permanently stored, ensuring long-term availability and verifiability (42); and (v) Censorship Resistance: Both technologies together enhance the system's resistance to censorship (43). Data is not stored in a single location, and any single entity does not control the data.

#### 2.1.3 AI diagnosis

There has been considerable development in using AI models to diagnose oral health status as part of monitoring oral health remotely. A systematic review of AI models in diagnosis reported accuracy for detecting dental plaque in the range of 74%–99%, the accuracy of intraoral photographs for diagnosis of gingivitis as between 74% and 78%; the use of fluorescent intraoral images to diagnose gingivitis with an accuracy of between 68% to 74%; intraoral photographs in the diagnosis of periodontal disease's accuracy was lower between 47% and 81% (42, 44). A later study reported AI models for gingivitis screening using intraoral photographs with sensitivity and specificity up to 92% and 94%, respectively (45), and the latest one reported a pilot study within real-world communities with up to 93% sensitivity (46).

Ensuring the “accuracy” and “reliability” of AI algorithms used for diagnoses is critical to prevent misdiagnoses and incorrect treatment recommendations (47). Inaccurate diagnoses can lead to incorrect treatment plans. This can harm patients, including unnecessary procedures, delayed treatment of the condition, and/or inappropriate procedures. Inaccurate or unreliable AI diagnoses can erode trust in the technology and in the healthcare system. As such, providers must validate AI systems and continuously monitor their performance to maintain high levels of accuracy ethically before they are applied in practice (48). Patients should have the right to seek a second opinion from a human healthcare provider and not solely rely on an AI-generated diagnosis.

AI algorithms can exhibit biases based on the data they are trained on, leading to disparities in diagnoses for certain patient populations, for example often underserved communities whose data is not part of training set (49). Biased diagnoses may disproportionately affect certain groups based on race, gender, age, or socioeconomic status (50). Biased algorithms can exacerbate existing health disparities by providing less accurate diagnoses for underrepresented or marginalized populations, leading to unequal access to quality care (51). Addressing bias in AI diagnoses is challenging but essential to ensure fairness, equity, and non-discrimination in healthcare delivery.

AI through machine learning has also been applied and tested for caries diagnoses (50, 52), maxillary sinus diseases (53), salivary gland diseases (54), TMJ disorders (55), and oral cancer (56) through clinical data and diagnostic images with varying degrees of accuracy and similar ethical concepts.

AI systems often operate as black boxes, making it challenging to understand how they arrive at a particular diagnosis (57). Ensuring “transparency” and “explainability” in AI diagnoses is crucial for healthcare providers to trust and interpret the results generated by these systems (58).

Moreover, determining “accountability” for AI-generated diagnoses is complex, especially when errors or adverse outcomes occur (59). Regulatory bodies must work towards establishing clear guidelines for oversight, regulation, and accountability in AI diagnoses - holding the healthcare providers, users and AI developers responsible for their decisions and/or the healthcare systems that use these types of AI. Legal frameworks must also address “liability” issues where AI-driven diagnoses harm patients. This includes clarifying the roles and responsibilities of all stakeholders involved.

While AI can arguably enhance diagnostic accuracy and efficiency, it should not replace human judgment and expertise. Maintaining human oversight and collaboration in AI diagnoses is crucial to ensure healthcare providers can interpret AI-generated results, provide context, and make informed treatment decisions (60). Addressing these ethical issues in AI diagnoses requires a multidisciplinary approach involving dentists/healthcare providers, AI developers and policymakers of healthcare systems. By prioritizing these ethical considerations, we can maximize the benefits of AI technologies while upholding patient safety, privacy, and trust.

AI and deep learning technologies have long been established in orthodontics for treatment planning (61). For example, artificial neural networks can detect the need for tooth extraction and determine what teeth are needed for orthodontic treatment. Success rates in diagnosing extractions have been reported to be in the 90% range. This has implications, particularly in assisting the more junior, inexperienced clinicians (62).

Support during treatment via computer-based decision support systems has also been applied, such as monitoring tooth root locations during orthodontic treatment to prevent unfavourable outcomes and enhance treatment success (63). Moreover, sequential images can assist with monitoring the progress of orthodontic treatment and ensure greater treatment effectiveness by reducing the unnecessary frequent visits by patients in person in surgery (64). However, current scoping review results suggest that widespread clinical use of end-to-end machine learning tools is still a distant goal (61).

Remote monitoring in orthodontics involves using digital technologies to track and manage patients' progress outside of traditional in-office visits (65). While this approach offers several benefits, it also raises several ethical issues that need to be carefully considered, such as (i) Privacy, Patient Confidentiality and Data Security (66); (ii) Voluntary participation - the option to opt out of remote monitoring without facing any negative consequences or reduced quality of care (67); (iii) Relying heavily on remote monitoring may lead to a reduction in the quality of care if it replaces necessary in-person evaluations (68); (iv) Ethical issues associated with excess/over-monitoring, where patients may feel overwhelmed by the constant attention to their progress and deviations in treatment (69); and (v) Clear communication about the role and limitations of remote monitoring to help maintain trust and manage patient expectations (70).

Addressing these ethical issues requires a balanced approach that prioritizes patient welfare, privacy, and autonomy while leveraging the benefits of remote monitoring to improve access and convenience in orthodontic care (71). This involves ongoing evaluation, transparent communication, and adherence to ethical and regulatory standards.

### 2.2 Digital technologies and devices

#### 2.2.1 Wearables

Wearable technology has become increasingly popular for monitoring various aspects of health (72). These devices have sensors that track various biometric data, providing users and healthcare providers valuable insights into health status and trends (73). These have been used widely in medicine, from temperature and hydration monitors to fitness trackers.

They are also adapted in dentistry: (i) Smart toothbrush sensors that monitor brushing habits, track brushing duration and pressure, and provide real-time feedback via a connected app to improve brushing techniques (74); (ii) Smart Retainers and Aligners can monitor wear time and treatment progress. Some may have built-in sensors that communicate with a smartphone app to track usage and ensure patients follow their treatment plans correctly (75); (iii) Wearable Intraoral Sensors & Wearable Biosensors to monitor oral health parameters, such as pH levels, bacterial activity, and specific biomarkers related to oral diseases (76); (iv) Wearable cameras to capture high-resolution images or videos inside the mouth, which can be helpful for remote dental consultations, monitoring treatment progress, or detecting early signs of oral health issues (77); (v) Smart dental implants can monitor the health and stability of the implant site, detect early signs of infection, and provide data on the load and stress the implant is experiencing (78); and (vi) Oral Health trackers, for example, night guard monitoring of clenching and grinding (79).

These wearable technologies can enhance patient engagement, improve treatment outcomes, and facilitate better communication between patients and dental professionals (80). However, it also raises a whole range of ethical issues. Wearable Devices store large amounts of personal information accessed by third parties without user consent (81). This creates ethical issues regarding privacy, security and informed consent.

Related to this is the remote use of oral health data by way of Computer-assisted design and manufacturing (CAD-CAM) technologies that have rapidly expanded in dentistry in recent decades (82–84), with CAD-CAM intraoral crowns and prostheses being milled, printed, or sintered such that replacing traditional technician-dentist delivery models (85). The turnover of new technologies is rapid and diverse, with an almost seamless data exchange facilitated by open data file formats.

The issue of replacement of this CAD-CAM prosthesis will need to be replaced eventually, and there have been calls to establish best practices and protocols, including a clarification of the “blueprint” ownership, which has legal and ethical considerations (86).

A further complication is that in many countries, the legal responsibility is placed firmly on the dentist to ensure an appropriate design of the intraoral device, including the choice of biomaterials and their handling (87). In contrast, the production device today may be operated, located in a different country, and applicable to their respective national law. Hence, CAD files today can be anywhere and nowhere, although still subjected to governmental patient privacy regulations in all countries involved (87, 88).

An editorial in clinical experimental dental research suggests that because the dentist is legally responsible for what enters the mouths of patients, it seems prudent that at least a copy of the “blueprint” of the intraoral device is retained in the patient records for documentation (86). It also seems prudent that dentists refrain from giving carte blanche to the designer of the intraoral device or to the production device centre to proceed with refabricating an intraoral device from an old blueprint before the dentist has provided input or approved this blueprint.

A scoping review identified almost 100 App studies related to Mobile Phone Apps for Oral Health, with monitoring oral health remotely often as a key feature (89). Most studies (31/45, 69%) concerned oral health promotion using mobile phone apps, followed by behaviour management – dental anxiety (5/45, 11%). More than half of the studies were conducted in Asia, and approximately a third were among adolescents. Among RCT, approximately 40% (9/23) reported a substantial reduction in dental plaque, and 26% (6/23) of the studies reported significant improvement in gingival health. Regarding dental anxiety management, 13% (3/23) of the RCT studies reported a significant decrease in mean heart rate and lower Facial Image Scale anxiety scores.

As previously mentioned, accessibility and inclusivity are potential ethical issues, particularly informed consent, given that many apps are aimed at adolescents. There are also privacy and security issues, as we all share data. A major issue is the accuracy and reliability of Apps, as many have commercial interests at stake with little if any, professional oversight. There have been repeated calls for frameworks to build health apps and mHealth that have been devised in an ethical manner to promote their safer use (90). In medical literature, there has been particular concern relating to mental health apps.

#### 2.2.2 Gamification and health

Game-like elements have become increasingly popular in the context of health apps (91). Gamification uses techniques and elements of video game design in non-game contexts (92). A recent scoping review identified that there has been a rapid expansion of gamification apps for oral health among children and adolescents (93). A substantial portion, approximately three-quarters of the studies, discussed oral self-care apps supported by evidence-based oral health. The most clearly defined data are “brushing time” (11/11, 100%) and “daily amount brushing” (10/11, 91%). Most studies (11/15, 73%) mentioned oral healthcare behaviour change techniques. They included “prompt intention formation” (11/26, 42%), “providing instructions” (11/26, 42%), “providing information on the behaviour-health link” (10/26, 38%), “providing information on consequences” (9/26,35%), “modelling or demonstrating behaviour” (9/26, 35%), “providing feedback on performance” (8/26, 31%), and “providing contingent rewards” (8/26, 31%).

While such “gamified” apps hold great potential in motivating people to improve their health, they also potentially come with a “darker side”, as evidence from the medical literature outlines. (i) Gamification techniques can be highly engaging, and this can be particularly problematic if it leads to neglecting other vital aspects of health or life (94); (ii) The competitive nature of gamified health apps can have mixed effects on users' mental health, from motivating to stress, anxiety, or decreased self-esteem if they are unable to meet goals or compare unfavourably to others (95); (iii) Gamification techniques are designed to influence user behaviour so these techniques must be used ethically and not to manipulate users in ways that could be coercive or harmful (96). It is also important to remember that while gamification can be effective in the short term, its long-term efficacy in promoting sustained health behaviour change is unclear (97). It is important to consider whether the benefits are lasting or if they diminish once the novelty wears off.

#### 2.2.3 Chatbots

AI chatbots have transformed digital communication with the potential to enhance interactions significantly (98), and this has the potential for use in answering questions and giving feedback on oral health remotely. Leveraging deep learning algorithms, these chatbots are trained on extensive datasets and continuously refine their response accuracy and relevance by mimicking human neural networks (99). Leading platforms in this domain include Chat GPT by OpenAI Inc., Google Gemini by Google LLC, Bing by Microsoft Corporation and Claude from Anthropic PBC. Their introduction into medicine and dentistry has the potential to optimize resource utilization and reduce the need for extensive manpower to answer common questions, making medical information more accessible to the general public (100). Are they valid and reliable enough to do so, and what are the ethical considerations?

At the end of 2024, Dental Traumatology published a paper reporting on the validity and reliability of these four commonly used AI chatbots in addressing frequently asked questions related to dental trauma (20 questions compared with clinical experts) (101). In terms of reliability, there were overall acceptable levels; however, in terms of validity, there were considerable variations at both low and high threshold levels.

This raises the issues of misinformation and deception, which has been termed an “AI-driven infodemic”, a public health threat from using chatbots to produce a vast amount of scientific articles, fake news, and misinformative content (102). The “AI-driven infodemic” results from their ability to quickly write large amounts of human-like texts, not only with malicious intent but in general without any scientific ground and support.

To address this public health threat is important to raise awareness and rapidly develop policies through a multidisciplinary effort, such as updating the current WHO public health research agenda for managing infodemics. There is a need for policy action to ensure that the benefits are not outweighed by the risks they pose. In this context, it is proposed to introduce a detectable-by-design approach, which involves building LLMs with features that make it easier to detect when they are being used to produce fake news or scientific articles. However, implementing this approach could slow down the development process of LLMs, and for this reason, AI companies might not readily accept it.

There is no formal consensus on the safe and ethical implementation of AI systems in healthcare settings, but the literature converges on several key principles of ethical AI use including transparency, justice and fairness, non-maleficence, responsibility and privacy (103). There have been calls for the constitution of groups of experts inside health international agencies (e.g., WHO, ECDC) dedicated to monitor the use of LLMs for fake news and scientific articles production is needed, as the scenario is rapidly evolving and the AI-driven infodemic threat is forthcoming. Such groups could work closely with AI companies to develop effective strategies for detecting and preventing the use of LLMs for nefarious purposes.

## 3 Conclusion

Remote health monitoring combines on-demand and implicit monitoring (via wearables and smart devices) of oral health, helping dentists and other healthcare practitioners gain ongoing visibility of patient data that can signal changes in the patient's oral health condition, assist in diagnosis, provide advice, education and oral health-promotion, treatment decision and care pathways, actively inform ongoing treatment and provide follow-up evaluations and post-discharge monitoring.

It has many potential advantages for improving the effectiveness and efficiency of dental/oral health care, such as close engagement with patients by monitoring patient health remotely; earlier oral disease detection and associated better treatment outcomes; a reduced number of visits reduces barriers for patients with mobility issues and other access issues; reduced number of readmissions; it helps in social distancing and assists in addressing the shortage of trained health professionals, especially in underserved regions. Nevertheless, there are still many ethical issues and limits in the ethical framework that guide us.

## Data Availability

The raw data supporting the conclusions of this article will be made available by the authors, without undue reservation.
